# Drug Delivery Systems as a Strategy to Improve the Efficacy of FDA-Approved Alzheimer’s Drugs

**DOI:** 10.3390/pharmaceutics14112296

**Published:** 2022-10-26

**Authors:** Débora Nunes, Joana A. Loureiro, Maria Carmo Pereira

**Affiliations:** 1 LEPABE—Laboratory for Process Engineering, Environment, Biotechnology and Energy, Faculty of Engineering, University of Porto, Rua Dr. Roberto Frias, 4200-465 Porto, Portugal; 2 ALiCE—Associate Laboratory in Chemical Engineering, Faculty of Engineering, University of Porto, Rua Dr. Roberto Frias, 4200-465 Porto, Portugal

**Keywords:** Alzheimer’s disease, memantine, rivastigmine, donepezil, galantamine, nanoparticles, hydrogel, nanoparticle-loaded hydrogel, nanomaterials, drug release

## Abstract

Alzheimer’s disease (AD) is the most common form of dementia, with a high impact worldwide, accounting for more than 46 million cases. The continuous increase of AD demands the fast development of preventive and curative therapeutic strategies that are truly effective. The drugs approved for AD treatment are classified into acetylcholinesterase inhibitors and N-methyl-D-aspartate receptor antagonists. The therapeutic effectiveness of those drugs is hindered by their restricted access to the brain due to the blood–brain barrier, low bioavailability, and poor pharmacokinetic properties. In addition, the drugs are reported to have undesirable side effects. Several drug delivery systems (DDSs) have been widely exploited to address these issues. DDSs serve as drug carriers, combining the ability to deliver drugs locally and in a targeted manner with the ability to release them in a controlled and sustained manner. As a result, the pharmacological therapeutic effectiveness is raised, while the unwanted side effects induced by the unspecific distribution decrease. This article reviews the recently developed DDSs to increase the efficacy of Food and Drug Administration-approved AD drugs.

## 1. Introduction

Alzheimer’s disease (AD) is a neurodegenerative disease responsible for about 75% of global dementia cases [1]. In 2040, the incidence of AD is anticipated to reach 140 million people [2]. Dementia is now predicted to cost $1 trillion yearly worldwide. This number includes direct, indirect, and intangible costs. Direct costs involve health care and paid social care. Indirect costs, which are frequently overlooked, comprise informal care provided by close people and the patient’s inability to work, reducing their productivity. Lastly, intangible costs include patients’ and caregivers’ reduced quality of life [3]. The ever-increasing prevalence of AD demands the rapid development of effective therapeutic strategies. Histologically, AD is characterized by the appearance of amyloid plaques and neurofibrillary tangles (NFTs) in the brain [4,5]. Amyloid plaques are deposits of the β-amyloid (Aβ) peptide, while NFTs result from hyperphosphorylation of the tau protein. Clinically, these characteristics lead to memory loss, disorientation, cognitive and motor impairment, and aggressive behavior [6].

The pharmacologic therapies for AD are classified into two classes: acetylcholinesterase (AChE) inhibitors and N-methyl-D-aspartate (NMDA) receptor antagonists [7]. However, AChE inhibitors and NMDA receptor antagonists have been linked to side effects. In addition, the drug’s therapeutic effectiveness can be limited by the biological barriers that prevent drugs from reaching the brain and by their inherent poor properties, such as low bioavailability and poor pharmacokinetics and pharmacodynamics.

Drug delivery systems (DDSs), such as nanoparticles (NPs), hydrogels, microformulations (microneedles, microparticles, microspheres, and microemulsions), and NP-loaded hydrogel (NLH) systems, have been widely employed to address these issues. NPs and microparticles can improve drugs’ therapeutic efficacy by protecting them from degradation, enhancing their bioavailability, and allowing for a more sustained and localized release [8,9]. Additionally, their surface could be functionalized for a targeted administration, overcoming the biological barriers and allowing a drug release in the target tissue. Thus, the undesired side effects induced by the unspecific distribution over the different tissues will be reduced [9]. Hydrogels are porous structures with a high water retention capability and solute permeability [10,11,12,13]. They can effectively encapsulate drugs, protecting and releasing them over time, while raising their local concentration and decreasing their toxicity in the remaining tissues [14]. The NLH systems incorporate NPs into hydrogels and have emerged to improve their performance compared to each alone. It is feasible to combine the target delivery of the NPs with the local delivery of the hydrogels, allowing the drug uptake in the required location. In addition, as both provide a controlled and sustained release, the final system synergizes drug release patterns, resulting in improved therapeutic effectiveness.

In this regard, this review aims to discuss the most recently developed DDSs for Food and Drug Administration (FDA)-approved Alzheimer’s drugs, emphasizing there in vitro and in vivo performance. Due to the relevance of this topic to the scientific community, several review papers aiming at the use of NPs as DDSs for AD management have been published in recent years [15,16,17,18,19,20]. Despite the extensive information provided, those works only addressed the use of NPs. Thus, the current work provides the first review addressing not only the use of NPs but also the use of hydrogels, microformulations, and NLH systems as DDSs to improve the efficacy of AD drugs, and comprises all the research data published between 2012 and 2022. This review highlights the benefits of the existing DDSs for FDA-approved Alzheimer’s drugs.

## 2. Pharmacological Therapeutic Strategies for AD

**Figure 1:**
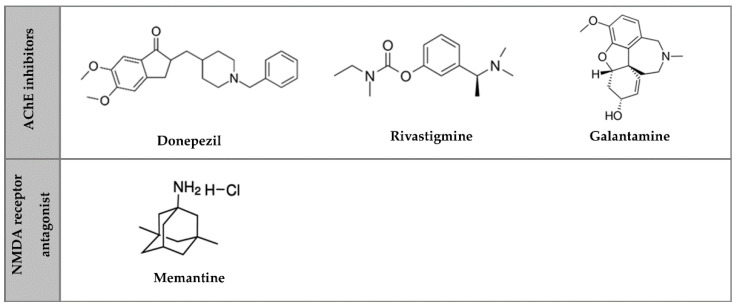
Chemical structure of AD drugs.

Currently, there are no treatments to stop or reverse AD since the disease leads to the death of brain cells, an irreversible process, and loss of brain tissue [3], which has no regeneration capacity. There are only symptomatic treatments, with drugs used to maintain the patient’s quality of life, improve their cognitive functions and decrease disease progression [21]. Those pharmacological treatments are classified into AChE inhibitors and NMDA receptor antagonists (Figure 1) [21].

AChE is an enzyme that degrades acetylcholine, a neurotransmitter involved in synapses. AChE inhibitors block AChE, causing acetylcholine accumulation, activating nicotinic and muscarinic receptors, and disturbing neurotransmission [22]. The AChE inhibitors approved by FDA and European Medicines Agency for AD treatment are donepezil for mild to severe AD, and galantamine and rivastigmine for mild to moderate AD [22,23]. Donepezil, commercially available as Aricept^®^, is available in tablet form and administered in a daily dose range of 5 to 10 mg. In March 2022, the FDA approved the first transdermal system for donepezil administration in patients with mild, moderate, and severe AD: the Adlarity^®^. The patch is a once-weekly transdermal formulation for a consistent dose administration of donepezil, available in 5 or 10 mg/day formulations [24]. Galantamine, commercially known as Reminyl^®^ and Razadyne^®^, is available in tablets, capsules, and oral solutions, and is administered daily to 16 to 24 mg [25]. Rivastigmine is commercialized as Prometax^®^ (tablets) and Exelon^®^ (capsules, oral solution, and transdermal patch). Its daily dose range is 6 to 12 mg when orally administered or 9.5 mg when transdermally administered [22,26,27].

The NMDA receptor is a glutamate ligand, the brain’s principal excitatory neurotransmitter. Glutamate may induce excitotoxicity, causing AD. This receptor performs a critical role in brain plasticity, synapse structure, survival of neurons, cognitive functions, and the establishment of long-term memory [23,27]. Memantine is currently the only NMDA receptor antagonist approved and is used in moderate to severe stages of AD [23]. Namenda^®^ is a brand name for memantine, sold as tablets and oral solutions. Memantine’s dose range is about 5 to 20 mg/day [26].

Since the underlying mechanisms of AD are still unknown, a combined treatment using both AChE inhibitors and NMDA receptor antagonists for moderate to severe AD has been recommended. As they have different mechanisms of action, it is possible to take advantage of their benefits in one treatment [27]. This mixture is achieved using memantine and donepezil, commercialized as Namzaric^®^.

In 2021, the FDA approved aducanumab (Aduhelm™), a monoclonal antibody that binds to Aβ oligomers and promotes their clearance, considered the first demonstrated therapy that reduces amyloid plaques. This drug is administered by intravenous infusion in four doses at 4-week intervals. Despite its promised effects, there is currently inadequate evidence to assess its efficacy as an AD cure [28].

## 3. Shortcomings of AD Pharmacological Therapies

Pharmacological therapies face several challenges that compromise their effectiveness. Oral pills, oral solutions, and transdermal patches are the current forms of AD drugs. Besides the easy application, safety, convenience, and economic advantages of oral administration, some limitations compromise the therapeutic effect. The drug concentration is significantly reduced after oral administration until it reaches its action site. The first pass metabolism, a liver-related absorption mechanism, is primarily responsible for this reduction [29]. Another drawback is poor drug targeting [30], which slows their onset of action and induces systemic toxicity. In addition, biological barriers are the main impediment to most drugs reaching the central nervous system (CNS), leading to a lack of brain targeting [30]. Due to drugs’ low solubility, permeability, and incompatible molecular weight or charge, their capacity to cross the blood–brain barrier (BBB) is restricted, decreasing drug concentrations in the brain and reducing the therapeutic effect of its administration [31,32]. These challenges lead to higher dosages and more frequent administrations to reach effective doses and maintain the plasma levels within the therapeutic window, which induces toxicity in the remaining tissues due to exposure to peripheral organs and undesirable side effects [33,34]. The higher dosages can also cause drug resistance in some instances.

The side effects may limit the ability of patients to take the drugs and hinder patient compliance. The most common side effects of AChE inhibitors include gastrointestinal symptoms such as dizziness, nausea, vomiting, and diarrhea [35,36]. While rare, syncope has also been correlated to using these drugs, which could be caused by arrhythmia. AChE inhibitors are widely known for causing sinus bradycardia [35,37]. For memantine, adverse reactions include dizziness, agitation, confusion, headache, diarrhea, and constipation [36,38]. Those side effects usually occur at the beginning of therapy, when higher dose ranges are used, and whenever the dose is increased.

### Possible Strategies to Overcome the Blood–Brain Barrier

**Figure 2:**
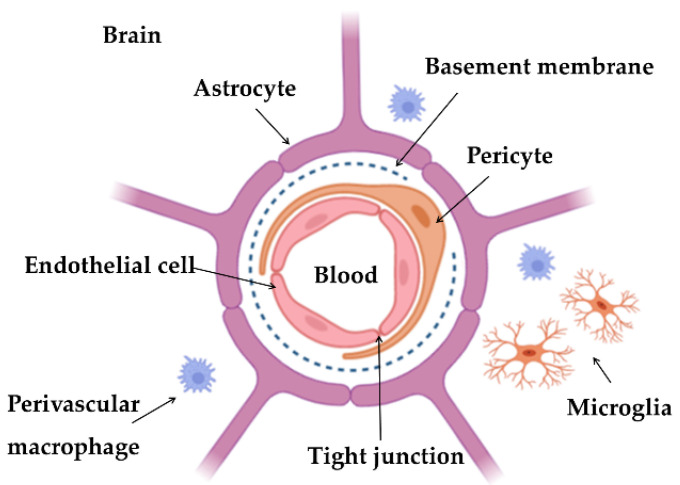
Illustration of BBB constitution.

The brain has three essential barriers that contribute to its homeostasis, strictly regulating the exchanges between the blood and the cerebrospinal fluid: (i) choroid plexus epithelium, separating the blood and ventricular cerebrospinal fluid; (ii) arachnoid epithelium, the boundary between blood and subarachnoid cerebrospinal fluid; and (iii) BBB, that constitutes the blood–brain interface [39,40]. BBB is a very complex barrier allowing only a few substances to reach the brain. BBB is composed of endothelial cells, basement membrane, astrocytes, and pericytes [41,42], as illustrated in Figure 2.

The endothelial cells are joined by tight junctions, mainly responsible for limiting the passage of substances, working like a diffusion barrier. These substances’ characteristics, such as charge, lipophilicity, and polarity, are also obstacles [43,44]. The mainly transport mechanisms by which the substances can cross the BBB include: (i) diffusion; (ii) transport proteins; (iii) efflux pumps that return the unwanted substances to the blood; (iv) receptor-mediated transcytosis; (v) adsorptive-mediated transcytosis, and (vi) cell-mediated transcytosis. Diffusion is divided into two types: paracellular, which is related to small molecules (with a mass lower than 150 Da) that diffuse across tight junctions, such as water; and transcellular, which is associated with small lipid molecules that diffuse passively through the cell (less than 400–600 Da). The second is a specific receptor-mediated or vesicular mechanism for molecules and substances essential to the CNS’s normal functioning, such as amino acids, glucose, or organic anions and cations. The transport proteins act as molecule carriers across the membrane from the side with a higher concentration to the side with a lower concentration. Receptor-mediated transcytosis is a mechanism for macromolecule transport across the endothelial cells. The macromolecule attaches to a receptor, and then a vesicle encapsulates the element and transports it across the membrane. Examples of those macromolecules include insulin, Tf, enzymes, and lipoproteins. Adsorptive-mediated transcytosis is based on electrostatic interactions between the membrane and the substance. Finally, cell-mediated transcytosis is the mechanism through which any type of molecules or pathogenic and infectious agents enter the brain, and is mediated by immune system cells, such as the monocytes [40,42,45,46].

According to the previously described BBB characteristics, this barrier is the most critical obstacle to many potential drugs for treating CNS diseases such as AD, preventing them from reaching the brain.

## 4. Drug Delivery Systems against AD

As a result of the significant breakthroughs in the biomaterials area, several DDSs have arisen to address the shortcomings of conventional drug delivery by enhancing the drug’s pharmacological characteristics. DDSs can improve the drug’s solubility and bioavailability, reducing drug degradation and lengthening its half-life [47,48]. DDSs can maintain a consistent drug concentration for an extended period due to its ability to release drugs in a controlled and sustained manner, preventing drug plasma level fluctuations. Additionally, DDSs can be tailored with suitable properties to overcome the BBB and provide targeted and localized therapy by releasing the drugs directly into the tissue where they must act. Then, the required dose to achieve a therapeutic effect is lowered, as well as the frequency of administration, which avoids systemic toxicity and undesirable side effects. Consequently, therapies become more accurate, effective, and less invasive [47,48,49,50].

### 4.1. Nanoparticles

NPs are colloidal structures composed of lipids, metals, natural polymers, or synthetic polymers [51]. For neurological applications, typically, their size is between 150 and 200 nm, a size range that would provide prolonged blood circulation and make them an effective vehicle for passage through the BBB [52]. The materials used to make NPs are critical because they affect the significant physicochemical properties of NPs, such as encapsulation rate, stability, and drug release pattern. Several types of NPs have already been produced with suitable properties to overcome the BBB and deliver drugs to the brain, in particular polymeric-based and lipid-based NPs, such as liposomes, solid lipid NPs (SLNs), and nanostructured lipid carriers (NLCs) [53,54,55,56]. NPs, through drug encapsulation, can protect drugs from chemical and enzymatic degradation. NPs can deliver drugs to a specific tissue by hiding their physicochemical properties while maintaining therapeutic activity [57,58]. Furthermore, NPs enable controlled and sustained drug release, allowing long-term maintenance of therapeutically active dosages. In addition, drug encapsulation into NPs reduces the required doses to produce the therapeutic effects due to an increased concentration at the location of action, reducing the associated effects [59]. Considering the multiple advantages, NP-based DDSs have a significant therapeutic potential for AD. The combined application of NPs and FDA-approved Alzheimer’s drugs undoubtedly provides promising options for AD treatment. Several approaches have been developed to achieve that, and their effects are described in the following subsections.

#### 4.1.1. Donepezil

Intranasal administration of NPs aids in the delivery of drugs into the brain by avoiding the BBB and decreasing systemic exposure, resulting in fast absorption and improved drug entry into the brain. For instance, Asmari et al. (2016) developed an intranasal approach for donepezil delivery using liposomes [60]. The liposomes were prepared by the traditional thin layer hydration method using 1,2-distearoyl-sn-glycero-3-phosphocholine (DSPC), cholesterol, and poly(ethylene glycol) (PEG). The in vitro donepezil release from the NPs was performed against the simulated nasal fluid. The results showed that liposomes increased the drug release for up to 8 h compared to the donepezil solution. In addition, the in vivo intranasal administration of donepezil-loaded liposomes significantly increased the drug levels in plasma and brain compared to oral or intranasal free drug administration, suggesting an excellent drug bioavailability following its nasal delivery using liposomes. No histopathological changes were found on the main organs following the intranasal administration of drug-loaded liposomes, demonstrating the safety of the drug carriers.

Similarly, Bhavna et al. (2014) proposed an intranasal donepezil administration through its encapsulation into chitosan NPs [61]. The NPs were prepared by the ionic cross-linking method, using tripolyphosphate as a cross-linker. The findings of the in vitro release assays showed that the drug was released in a sustained manner by the NPs. Further, to evaluate the in vivo performance, animal experiments were performed. In vivo safety tests revealed no hematological or histopathological changes, and no evidence of toxicity or of bodyweight differences in animals treated with the drug-loaded NPs. As a result, the intranasally administered NPs were considered safe. Furthermore, the drug content in the brain following intranasal delivery was estimated using pharmacokinetic in vivo studies. Compared to a drug solution, NPs increased the drug levels in the brain by nearly three times. The authors concluded that this NPs formulation increased drug concentration in the brain through a direct nose-to-brain delivery without causing toxicity.

The same authors proposed an intravenous approach for administering donepezil using poly(lactic-co-glycolic acid) (PLGA) NPs to enhance the drugs’ ability to cross the BBB, lowering side effects and improving therapeutic effects [62]. The NPs were prepared by the solvent emulsification–evaporation technique and coated with polysorbate 80 to increase their stability in order to successfully transport them across the BBB. The in vitro release pattern of donepezil-loaded NPs revealed an initial burst release followed by a controlled and sustained drug release for 25 days, whereas the donepezil solution showed total drug release in a few hours. The animals were treated intravenously with donepezil-loaded NPs and donepezil solution. Compared to the donepezil solution, donepezil-loaded NPs showed higher drug levels in the brain after intravenous administration, demonstrating that NPs can penetrate the BBB faster than the free drug. This increased drug concentration in the target tissue will improve the therapeutic outcomes of donepezil.

Another approach using polymeric NPs for intravenous administration of donepezil was proposed by Baysal et al. (2017) [63]. The carriers were composed of PLGA-block-PEG and produced with the double emulsion method. Donepezil was loaded into the NPs to minimize unspecific drug spread and gastrointestinal side effects while increasing patients’ compliance and treatment effectiveness. In vitro drug release revealed a controlled release profile of the donepezil from the NPs. In vitro, the destabilizing effect of donepezil-loaded NPs on Aβ peptide was examined, indicating an inhibition in fibril formation and aggregation. The ability of these NPs to cross the BBB was investigated in an in vitro BBB model employing human brain microvascular endothelial cells and human astrocytes with previously induced inflammation. The results indicate that the NPs successfully crossed the BBB, further supported by a reduction in inflammatory cytokine levels, meaning that donepezil had a neuroprotective impact.

Topal et al. (2021) proposed a strategy using lipid NPs to increase brain penetration of donepezil and reduce its side effects after intravenous administration [64]. The NPs were produced by the homogenization–sonication method and functionalized with apolipoprotein E (APOE) for brain targeting. While the donepezil solution reached a release of 85% after 8 h, the amount of donepezil released from SLNs after 72 h was 50%. As expected, APOE targeting ligand on the surface of SLNs significantly increased their cell uptake and permeability compared to the non-functionalized SLNs.

#### 4.1.2. Galantamine

Several studies on using NPs to manage AD by improving galantamine efficacy have recently been presented. For instance, Li et al. (2012) investigated, for the first time, the effects of intranasal administration of galantamine-loaded liposomes on AChE inhibition and galantamine pharmacokinetic behavior [65]. Liposomes were prepared using soya phosphatidylcholine, cholesterol, and propylene glycol (PG). In vitro cytotoxicity was performed with the drug solution, liposomes solution, and drug-loaded liposomes against rat pheochromocytoma PC-12 cells. Incorporating galantamine into liposomes, which were discovered to be non-toxic to cells, reduced the drug’s cytotoxicity. Further, studies were performed to evaluate the in vivo efficacy of the developed NPs. The animals were divided and administered with drug solution (orally), drug solution, liposomes solution, and drug-loaded liposomes (intranasally). The results revealed that galantamine-loaded liposomes offered increased drug bioavailability, superior pharmacokinetic behavior, and enhanced AChE inhibition.

Fornaguera et al. (2015) developed, for the first time, galantamine-loaded polymeric NPs to improve drug bioavailability and boost their therapeutic activity after intravenous administration [66]. The NPs were prepared with suitable properties for parenteral administration by the nano-emulsification method using PLGA as polymer and Tween^®^ 80 as a surfactant. In vitro studies revealed that NPs could retain 80% of the drug’s pharmacological activity following its encapsulation, as seen by the high AChE inhibition obtained compared to the drug solution. Two cell lines—HeLa cells and SH-SY5Y cells—were employed to determine the in vitro cytotoxicity, and results showed that NPs were non-toxic at the required therapeutic concentration. By controlling the drug’s pharmacokinetic parameters and minimizing its administration frequency, the authors concluded that NPs were appropriate for galantamine delivery.

Another approach to enhance galantamine bioavailability in the brain after oral administration was developed by Misra et al. (2016) [67]. The authors created SLNs as carriers for galantamine through the microemulsification method. The designed carriers were reported to improve the drug’s bioavailability almost twofold and modulate the drug’s pharmacokinetics properties. In vivo, behavioral studies were performed through the Morris water maze (MWM) test, which revealed a reversal of cognitive impairment, indicating the effectiveness of SLNs in memory recovery and behavioral achievement.

In a subsequent study, Sunena et al. (2019) synthesized thiolated chitosan NPs for intranasal administration and tested them in vivo [68]. The animals were treated with galantamine solution (intranasal and oral delivery) and galantamine-loaded NPs (intranasal delivery) for 7 days. Compared to oral and intranasal administration of galantamine solution, the NPs improved the pharmacodynamic behavior of the drug and the AChE levels. The results also demonstrate a considerable recovery in animals with induced amnesia produced by intranasal administration of galantamine-loaded NPs.

#### 4.1.3. Rivastigmine

Mohamadpour et al. (2020) developed polymeric NPs for brain delivery of rivastigmine through intravenous administration [69]. The NPs were produced with methoxy poly(ethylene glycol)-co-poly(ε-caprolactone) (MPEG-PCL). According to the findings, the NPs had an initial fast release, followed by a slow-release pattern over 8 h. In the in vivo tests, the animals were treated intravenously with rivastigmine-loaded NPs or with a rivastigmine solution as a control to assess the drug’s pharmacokinetic and pharmacodynamic parameters. The intravenously injected drug-loaded NPs offered better brain uptake clearance, higher drug concentrations in both brain and plasma, and longer drug half-life compared to the free drug. Furthermore, the pharmacodynamic assay revealed improved memory deficits and enhanced spatial learning in the animals treated with rivastigmine-loaded NPs.

Fazil et al. (2012) prepared chitosan NPs to improve the bioavailability of rivastigmine to the brain via intranasal delivery [70]. The NPs were efficiently prepared by the ionic gelation method using chitosan and sodium tripolyphosphate as a cross-linking agent. The in vitro release study demonstrated that the NPs slowed the drug release. In vitro permeability studies were tested in fresh nasal tissues extracted from porcine nasal cavities. The mucoadhesive properties of chitosan provide increased nasal residence, resulting in higher drug penetration through nasal mucosa when drug-loaded NPs are used. The pharmacokinetic parameters were assessed to determine the rivastigmine brain–blood ratio in vivo. The animal group intranasally administered with the drug-loaded NPs presented a higher concentration of rivastigmine in the brain than the other groups. The higher ratio reveals a direct nose-to-brain transport across the BBB, demonstrating the advantage of NP-mediated rivastigmine administration.

Rompicherla et al. (2021) evaluated rivastigmine’s pharmacokinetic and pharmacodynamic profile using different NPs [71]. Due to its direct access to the brain, the authors developed PLGA-NPs and liposomes to treat dementia through intranasal administration. In vivo experiments were performed to compare the effect of the drug-loaded NPs (intranasally) and the drug solution (intranasally and orally). The MWM and passive avoidance tests were used to assess the behavioral and cognitive improvements of acute and chronic dementia models. Compared to oral drug solution and drug-loaded NPs, liposomes revealed the best pharmacokinetic properties, with a lower clearance rate and high drug systemic bioavailability, mean residence time, and half-life. The liposomes also maintained the drug concentration in the plasma, preventing drug fluctuation levels. In addition, in rivastigmine-loaded liposomes, a relation was found between those pharmacokinetic properties and AChE inhibition. Furthermore, in both acute and chronic AD models, the memory deficit was considerably recovered in the presence of drug-loaded liposomes.

**Figure 3:**
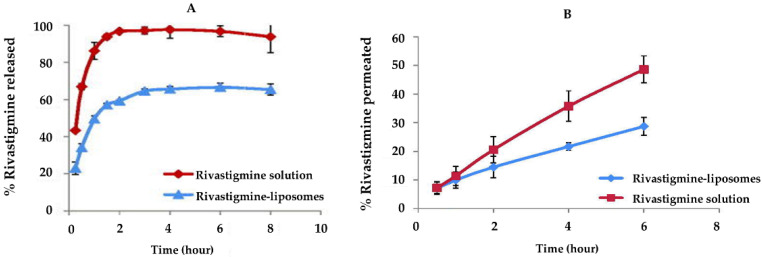
(**A**) in vitro release profile and (**B**) in vitro permeation profile of rivastigmine. Blue and red markers correspond to rivastigmine-loaded liposomes and rivastigmine solution, respectively. Adapted with permission from [72], copyright © 2017, published by Informa UK Limited.

Nageeb El-Helaly et al. (2017) also proposed rivastigmine-loaded liposomes for brain targeting through intranasal administration [72]. Liposomes were prepared by the thin-film hydration method, using PEG, 1,2-Distearoyl-sn-glycero-3-phosphorylethanolamine (DSPE), lecithin, didecyldimethyl ammonium bromide (DDAB), and Tween^®^ 80 as excipients. Compared to the drug solution, the in vitro release of rivastigmine from liposomes revealed that liposomes prolonged the drug’s release and modulated its release pattern (Figure 3A). Ex vivo nasal toxicity and permeation through nasal mucosa were estimated using the nasal cavity mucosa of a sheep. No histopathological alterations were verified on nasal tissue, proving the liposome’s safety. According to the permeation assay, liposomes as carriers delivered twice as much drug in the tissue compared with rivastigmine solution (Figure 3B). For the in vivo evaluation, two groups of animals were treated with an intranasal administration of rivastigmine-loaded liposomes or with a rivastigmine solution as a control. The animals treated with the drug solution revealed high drug plasma levels, and the drug-loaded liposomes demonstrated increased drug levels in both plasma and brain. As a result, the authors concluded that liposomes increased drug bioavailability and successfully carried it over the BBB.

Ismail et al. (2013) proposed liposomes for the subcutaneous administration of rivastigmine [73]. In vitro results revealed that the liposomes could significantly sustain the drug and prolong its release compared to the drug solution. In vivo, experiments demonstrate the memory impairment characteristics of AD. An acute toxicity study was carried out after subcutaneous administration of rivastigmine solution and rivastigmine-loaded liposomes to evaluate their safety upon administration to animals. No signs of toxicity were observed, confirming the carrier’s safety. Compared to the drug solution, the rivastigmine incorporated into liposomes decreased drugs’ toxicity, reducing drug side effects. In addition, the authors verified that, while the cognitive impairment and the AChE activity were improved with drug solution and drug-loaded liposomes, rivastigmine incorporation into NPs achieved a more pronounced effect.

Salimi et al. (2021) proposed a liposomal formulation for transdermal delivery of rivastigmine [74]. The prepared liposomes released 52% of rivastigmine in 24 h, while the rivastigmine solution was 100% released in 2 h. To assess the ability of the liposomes to increase drug permeation, an ex vivo permeability study was evaluated through rat skin. The authors verified that liposomes significantly boosted drug penetration compared to the rivastigmine solution. Over 90% of the rivastigmine-loaded liposomes went through the skin 48 h after topical treatment. A drug pharmacokinetic evaluation was conducted after treating rats with the rivastigmine-loaded liposomes and rivastigmine solution. The results demonstrated that liposomes increased drug permeation through the epidermis and caused a high drug concentration in the dermis, resulting in elevated rivastigmine absorption into the systemic circulation.

An approach using SLNs for the intranasal delivery of rivastigmine was proposed by Shah et al. (2015) [75]. In vitro and ex vivo diffusion studies revealed higher diffusion for drug solution than for drug-loaded NPs during the initial 2 h, which was the time required for the drug to diffuse out from the SLNs. After that, rivastigmine-loaded SLNs showed greater in vitro and ex vivo diffusion than the rivastigmine solution, indicating higher penetration of the nanocarriers in the tissue. Furthermore, no histopathological damage, toxicity, or cell necrosis was found, supporting the NP’s safety for intranasal administration.

Comparatively, Arora et al. (2022) prepared rivastigmine-loaded SLNs for enhanced intranasal delivery to the brain [76]. The SLNs were prepared using glyceryl monostearate and polysorbate 80. To simulate the in vivo nasal barrier, ex vivo permeation and nasal histopathology investigations of rivastigmine-loaded NPs through goat nasal mucosa were conducted. The findings revealed high flux and diffusion coefficients and the safety of the SLNs for intranasal delivery. In vivo performance of the NPs was analyzed, and the intranasal delivery of drug-loaded SLNs showed enhanced pharmacokinetic characteristics and higher drug bioavailability, resulting in increased plasma and brain drug concentrations. Additionally, in vivo histopathology supported the formulation’s safety.

Basharzad et al. (2022) proposed mesoporous silica NPs to improve rivastigmine brain delivery after intravenous administration [77]. The NPs were coated with polysorbate 80 as a targeting ligand. For 25 h, the NPs exhibited a sustained and controlled in vitro release of rivastigmine. In vitro cytotoxicity studies against PC-12 cells revealed that they are safe, with no significant changes in cell viability. Further, in vivo studies were performed to evaluate the efficacy of the developed DDS. The findings revealed that rivastigmine-loaded NPs significantly outperformed free rivastigmine in brain-to-plasma concentration ratio, brain uptake clearance, and plasma elimination half-life. Polysorbate 80-coated NPs had considerably higher drug brain levels, demonstrating the substance’s abilities to facilitate NPs’ entry into the brain.

#### 4.1.4. Memantine

**Figure 4:**
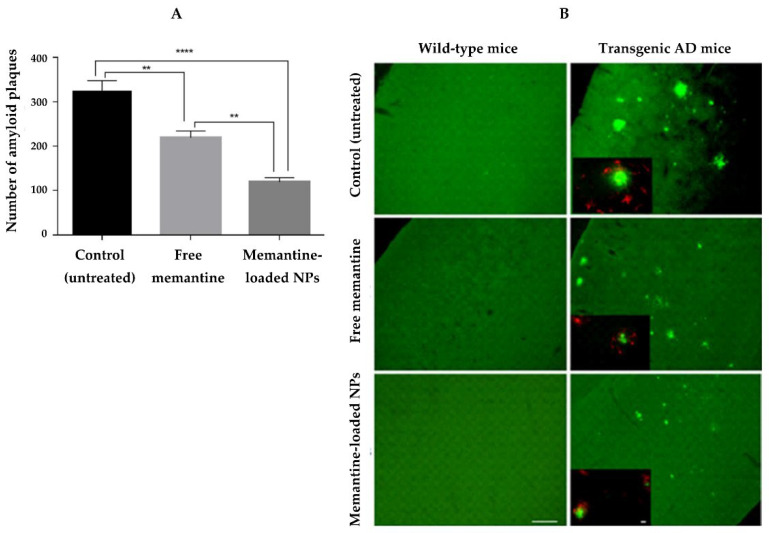
(**A**) Amyloid plaque counting. Data represent mean ± standard deviation. ** and **** Denotes statistically significant differences (*p* < 0.01 and *p* < 0.0001, respectively), and (**B**) immunohistochemical staining of amyloid plaques (green) and a primary antibody (red) of the cortex of wild-type and transgenic AD mice models. Scale bar: 100 µm. Adapted with permission from [78], copyright © 2018, Springer Nature.

To increase the effectiveness of memantine against AD, Sánchez-López et al. (2018) proposed PLGA-NPs as a carrier [78]. NPs were coated with PEG to enhance the mucus-permeating properties of NPs, increasing their bioavailability after oral administration. The in vitro release study proved that NPs released their content in a controlled and sustained way. In vitro cytotoxicity studies were performed on two brain cell lines (mouse microvascular endothelial cells and astrocytes from the brain rat cortex), where the NPs were found to be non-toxic. Furthermore, the transport through the BBB was observed in vitro and in vivo. Transgenic mice, which have mutations that allow them to secrete increased levels of human Aβ peptide, were employed as in vivo AD models. Using memantine incorporated in NPs, a higher reduction in memory impairment and improved learning abilities were observed. Furthermore, histological studies indicated that memantine-loaded NPs could reduce β-amyloid plaques and AD-related inflammation (Figure 4).

In another study for memantine delivery, Gothwal et al. (2019) synthesized poly(amidoamine) (PAMAM) dendrimers [79]. The dendrimers were conjugated with lactoferrin to increase their transport across the BBB. In contrast to the memantine solution, the in vitro release profile showed a prolonged and sustained release in the presence of dendrimers. The in vitro erythrocytic toxicity was studied using human blood, and the results revealed that lactoferrin decreased the hemotoxicity associated with the dendrimers. After intravenous administration of memantine solution, memantine-loaded dendrimers, and memantine-loaded lactoferrin-conjugated dendrimers, Sprague Dawley rats underwent in vivo pharmacokinetic experiments. In contrast, Swiss albino mice with Alzheimer’s disease underwent cognitive and behavioral tests. Lactoferrin-conjugated dendrimers increased drug bioavailability, concentration in the blood and brain, and half-life, extended residence time, and reduced the drug volume of distribution and clearance. The DDS showed a considerable improvement in memory and behavioral reactions during the cognitive assessments.

Table 1 provides an overview of the most recent applications of NPs as carriers for delivering FDA-approved Alzheimer’s drugs. The different types of NPs, their loaded cargo, the composition of NPs in terms of materials, the route of administration, and the main outcomes observed are highlighted in the table.

### 4.2. Hydrogels

Hydrogels are three-dimensional structures with excellent solute permeability and a high water retention capability [10,11,12,13]. Hydrogels can be biocompatible, biodegradable, and have low toxicity depending on the polymers used in their manufacture. Hydrogels can be formulated from a wide range of natural polymers, such as alginate, chitosan, gelatin, collagen, hydroxypropyl methylcellulose (HPMC), and hyaluronic acid; as well as synthetic polymers, which include polyacrylamide, poly(hydroxyethyl methacrylate), polyvinylpyrrolidone (PVP), poly(vinyl alcohol) (PVA), PEG, and poly-ε-caprolactone (PCL) [10,80]. Hydrogels can successfully encapsulate molecules, protecting and releasing them over time [14]. Due to their potential to respond to an external physical stimulus, thermosensitive hydrogels attract particular attention. The viscosity of thermosensitive hydrogels increases above physiological temperatures, allowing for liquid injection and in situ gelation [81]. As hydrogels are locally applied, they can boost drugs’ local concentration and lower their toxicity in the remaining tissues [14].

An approach to improving galantamine’s therapeutic effect was proposed by Rajkumar et al. (2022) [82]. The authors created an intracerebroventricular injectable hydrogel made of methacrylate gelatin. The animals’ behavioral activities under treatment with the hydrogel were evaluated in vivo in AD models. The galantamine-loaded hydrogel-treated rats showed improvements in body weight and blood glucose control, essential variables in developing cognitive skills. The role of these variables was then validated in the same animals, which demonstrated higher behavioral and cognitive activities compared to the control groups. Animals also showed a decrease in both Aβ aggregation and AChE levels. In addition, more significant activities of enzymes that decrease the effect of oxidative stress were observed, suggesting the neuroinflammation and antioxidant benefits of the drug-loaded hydrogel. In addition, histopathological and immunohistochemical studies revealed that the animals’ biochemical activity was significantly higher than that of controls.

Similarly, Bashyal et al. (2020) proposed using a hydrogel-based DDS for the transdermal administration of donepezil [83]. The hydrogel was composed of PVA and PVP. PG was added to the hydrogel as a chemical permeability enhancer across the skin. Its effect on donepezil permeation was investigated in vitro using skin obtained from Sprague Dawley rats. The results demonstrated that the PG significantly impacts donepezil permeability, which increases as the amount of polymer increases. To evaluate the in vivo performance of the hydrogel for transdermal delivery of donepezil, experiments were carried out in hairless rats. The animals were divided and separately treated with drug-loaded hydrogel (transdermally) and drug solution (intravenously). The in vivo pharmacokinetic studies revealed that plasma concentrations of donepezil were considerably higher for the animals transdermally treated with drug-loaded hydrogel. Furthermore, the plasma concentration of the drug was found to be dose-dependent on the donepezil concentration in the hydrogel.

Gu et al. (2020) took advantage of the thermosensitive properties of poloxamer 407 and poloxamer 188 to develop an in situ hydrogel for the intranasal administration of donepezil [84]. The hydrogel was prepared with the aforementioned polymers in combination with hydroxypropyl-β-cyclodextrin as a permeation enhancer. Due to the short gelation time and appropriate gelation temperature and pH, the hydrogel formulation met the in situ intranasal administration criteria. Compared to the donepezil solution, the hydrogel formulation showed a sustained donepezil release pattern in vitro. In vivo pharmacokinetics and brain targeting studies compared the administration of the drug loaded into a hydrogel (intranasal route) and drug solution (oral route). The main pharmacokinetic parameters in both plasma and the brain were significantly different between the formulations, revealing a better drug distribution to the brain when administered via the hydrogel. The drug’s bioavailability and targeting effectiveness significantly increased following hydrogel intranasal administration.

Table 2 summarizes the most recent applications of hydrogel as a DDS for delivering FDA-approved Alzheimer’s drugs, emphasizing the hydrogel’s composition, the drug incorporated in the hydrogel, the route of administration, and the in vivo and in vitro performance of the developed DDS.

### 4.3. Microformulations

**Figure 5:**
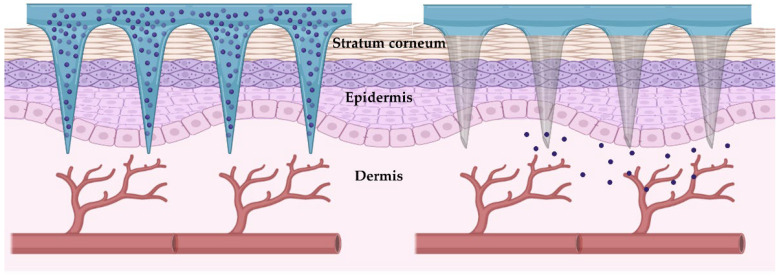
Schematic representation of the hydrogel-forming microneedles’ application and release.

Aside from the benefits, the skin barriers, especially the stratum corneum, could limit transdermal drug administration, hindering drug distribution across the skin. Strategies to improve skin permeability are extensively studied, with hydrogel-forming microneedles showing particular promise. Microneedles are micro-extensions of the transdermal patch base. They are designed to completely penetrate the skin and release the embedded molecules directly in the dermis, overcoming the skin barrier properties [85], as illustrated in Figure 5. Different microneedles—dissolving, solid, hollow, and coated microneedles—could be employed based on their configuration and the local drug entrapment. While the dissolving microneedles are made of biodegradable polymers and dissolve themselves over time, releasing the drugs, the others are removed intact and discarded after releasing their content [86].

Different approaches have been developed using hydrogel-forming microneedles. For example, Kim et al. (2016) proposed a transdermal hydrogel-forming microneedle for donepezil delivery [87]. The hydrogel base was produced with carboxymethyl cellulose, and the dissolving microneedles were prepared with HPMC. The insertion of hydrogel-forming microneedles into porcine skin and the amount of drug transport across the skin were studied in vitro. The results revealed that up to 78% of the drug could be encapsulated in the microneedles with enough mechanical strength to allow their successful skin insertion. In 15 min, the microneedles were completely dissolved in the skin. Over 95% of donepezil was released 5 min after microneedle insertion, resulting in the efficient delivery of the drug. Sprague Dawley rats were used in the in vivo studies. The animals were separately treated with donepezil-loaded hydrogel-forming microneedles and oral donepezil solution as a control. The animal’s plasma levels of donepezil were measured. When compared to oral administration, the hydrogel-forming microneedle enhanced the donepezil bioavailability. The authors concluded that using these DDSs can replace the oral administration of donepezil and improve patients’ compliance, resulting in more effective and convenient treatment.

Kearney et al. (2016) also developed a microneedle-mediated delivery of donepezil [88]. The DDS was a transdermal patch consisting of hydrogel-forming microneedles combined with a donepezil reservoir. In contrast to dissolving microneedles, the drug reservoir is designed separately from the microneedles. With this system, the amount of drug loading is not constrained by the size of the microneedles, and the drug release may be controlled more precisely. The hydrogel microneedles were prepared with copolymers of poly (methyl vinyl ether and maleic anhydride (PMVE/MAH)), PEG, and sodium carbonate. At the same time, the donepezil film was designed with PVP and glycerol. In vitro penetration studies of donepezil into neonatal porcine skin found that the DDS significantly increased drug penetration compared to the control. Sprague Dawley rats were used for in vivo pharmacokinetic analysis. In addition, plasma concentrations were noticeably greater following the administration of the hydrogel-forming microneedles to the animals.

**Figure 6:**
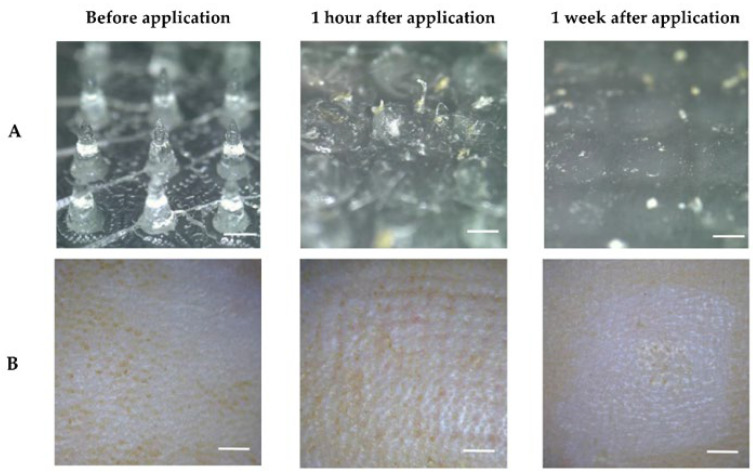
(**A**) In vivo dissolution of microneedles over time. Scale bar: 500 µm, and (**B**) in vitro skin irritation of donepezil-loaded hydrogel dissolving microneedles. Scale bar: 2 mm. Adapted with permission from [89], copyright © 2021 MDPI.

Similarly, Rehman et al. (2022) synthesized a hydrogel as a transdermal administration of donepezil via dissolving microneedles [89]. The hydrogel base was produced with polydimethylsiloxane, and the microneedles with PVP. In vitro studies revealed that the microneedles were gradually degraded (Figure 6A), allowing for the sustained release of the drug. Additionally, in vitro skin irritation studies showed no irritation response (Figure 6B). Further, in vivo experiments were performed, and the findings corroborated with the in vitro data, showing a complete dissolution of the microneedles and a sustained release and consistent absorption of donepezil. The DDS increased the half-life of donepezil compared to oral and subcutaneous administration, reducing the frequency of drug administration. Furthermore, the convenient self-administration of the hydrogels can address the shortcoming of low patient compliance.

A similar approach for memantine administration was proposed by Vos et al. (2020) [90]. The hydrogel-forming microneedles were produced with polydimethylsiloxane (PDMS) and alumina. The drug reservoir was created with polylactic acid (PLA). The authors investigated how different factors affected drug diffusion, including the microneedles’ conformation, hydrogel thickness, size, reservoir volume, drug concentration, and release buffer volume. Based on the findings, a model was created to predict the release kinetics of memantine as a function of the properties of the DDS. The optimized formulation was transdermally administered to minipigs for in vivo pharmacokinetic analysis. The animals were treated for 72 h with a memantine-loaded DDS and a PBS-loaded DDS as control. The results revealed that the DDS delivered at least 9 mg of memantine in that time, a promising strategy for controlled transdermal delivery. Regarding skin tolerability, only slight erythema was observed after the DDS was removed, which entirely disappeared after 3 days.

The commercially available rivastigmine patches cause local skin irritation, rapid release, and drug losses [91]. To address these limitations, a transdermal approach for rivastigmine delivery was proposed by Guimarães et al. (2022) using hydrogel-forming microneedles [92]. The authors used alginate, k-carrageenan, and a mixture of both to produce three hydrogel-forming microneedles. The hydrogel made with alginate, combined or not with k-carrageenan, had the best transdermal delivery capabilities. Rivastigmine was incorporated into the microneedles, and despite the drug altering its mechanical properties, it could still penetrate the stratum corneum. Fresh porcine ear skin was used to assess the rivastigmine-loaded DDS in in vitro skin permeation. Skin permeation studies revealed that rivastigmine-loaded hydrogel-forming microneedles allowed for a more efficient controlled drug release than commercial patches. Further, the hydrogel-forming microneedles were harmless when removed, implying that they are safe to use.

Other microformulations comprise microparticles, microspheres, and microemulsions. Similar to NPs, those microformulations can protect drugs from degradation, improve their stability, be administered through different routes (enteral, parenteral, topical, or inhalation), and prevent side effects of drugs. Additionally, they can improve the efficacy and tolerability of drugs and patient compliance [9,93]. Research by Simon et al. (2016) developed a lactose inhalation spray-dried formulation based on microparticles for rivastigmine delivery [94]. The microparticles were prepared by spray drying from ethanol/water solutions containing rivastigmine with and without the incorporation of l-leucine at different proportions. L-leucine, a low-density amino acid with hydrophobic characteristics, is reported to improve spray-dried particles’ aerodynamic properties. The lactose inhalation spray-dried formulation was prepared with and without microparticles. In vitro, an aerosol deposition study was performed to estimate the pulmonary deposition of the formulations. The microparticle formulation presented better physicochemical characteristics, aerodynamic properties, and aerosolization performance than a lactose formulation, constituting a potential DDS for rivastigmine delivery.

**Figure 7:**
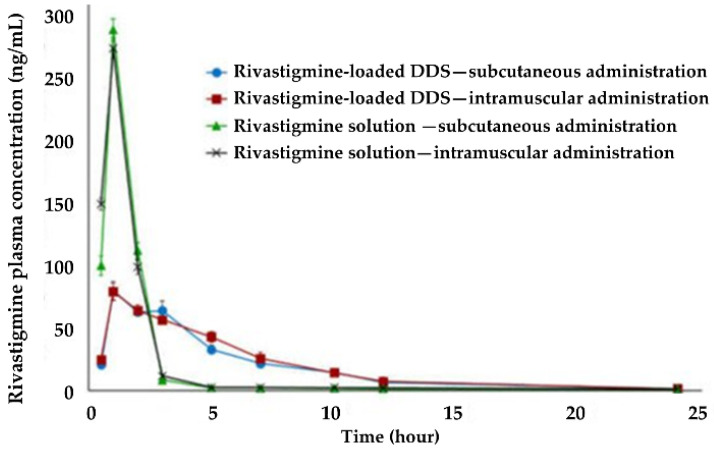
Rivastigmine plasma concentrations after in vivo administration in solution and through microparticles (mean ± standard deviation, n = 3). Adapted with permission from [95], copyright © 2021 MDPI.

Haider et al. (2021) developed in situ extended-release depots in the form of microparticles as a novel approach to improve the efficacy and tolerance of rivastigmine [95]. The polymeric microparticles were prepared using sucrose acetate isobutyrate (SAIB), PLGA, N-methyl-2-pyrrolidinone (NMP), and Pluronic^®^ F-68 by the emulsification method. The microparticles were then dispersed in an oily phase of sesame oil and sorbitan monostearate. In vitro cytotoxicity studies on bovine tracheal cells revealed the microparticles’ good biocompatibility and safety. In vivo, pharmacokinetic studies were assessed with albino rabbits. The animals were randomly assigned into four treatment groups, receiving a drug-loaded DDS or drug solution as control through subcutaneous and intramuscular injection. The outcomes revealed that microparticles extended the plasma levels of rivastigmine and increased its bioavailability. As expected, intramuscular administration produced higher plasma drug concentrations than subcutaneous injection, as muscles have better blood perfusion than subcutaneous tissue (Figure 7). In vitro, the release of the drug lasted about a month, which was not verified in vivo, and the authors concluded that further studies are needed to optimize the in vivo performance of the DDS’s release. The authors also concluded that additional research is necessary to improve the in vivo performance of the microparticles’ release, as the drug’s release lasted around a month in vitro but 1 day in vivo.

Gao et al. (2021) proposed a microspheres approach for nose-to-brain delivery of rivastigmine [96]. The microspheres were produced with ethyl cellulose, chitosan, and PVA, and their surface was modified with lectin to improve nasal adsorption. The produced microspheres’ rivastigmine release profile revealed a sustained release pattern with approximately full drug release within 12 h. The ex vivo bioadhesion study on goat nasal mucosa showed increased bioadhesion for lection-functionalized microspheres compared to non-functionalized microspheres. Following that, the microspheres’ performance was assessed in in vivo animal studies using Wistar rats administered with rivastigmine-loaded lection-functionalized microspheres through the nasal route and rivastigmine solution through nasal and oral routes. The animals treated with the DDS showed the best outcomes, with significantly improved memory retention, levels of biochemical parameters, and drug pharmacodynamics.

Microemulsions are colloidal DDSs with lipophilic components in combination with water and surfactants. These DDSs are reported to increase the penetration of drugs across biological membranes [97]. Recently, two studies have been presented on microemulsions as DDSs for delivering FDA-approved Alzheimer’s drugs. For instance, Shah et al. (2018) investigated the intranasal administration of rivastigmine using a microemulsion and a mucoadhesive microemulsion [98]. Both formulations were prepared by the water titration method using glyceryl caprylate, labrasol, transcutol-P, and water. For the mucoadhesive microemulsion, chitosan was added due to its well-known mucoadhesive characteristics. Animals received the microemulsions and rivastigmine solution intranasally, as well as microemulsions intravenously. High rivastigmine concentrations were detected in the blood and brain following intranasal administration of mucoadhesive microspheres compared to the remaining formulations. The findings demonstrated the effectiveness of chitosan in extending the microformulation retention in the nasal cavity, facilitating their nasal-to-brain transport, and boosting drug concentration at the target site.

In another study for intranasal administration, Espinoza et al. (2018) proposed using a microemulsion to improve donepezil’s effectiveness [99]. The donepezil-loaded formulation was successfully developed using castor oil, labrasol, transcutol-P, PG, and water, reaching a low viscosity and a pH value similar to the nasal mucosa, which prevents nasal irritation. In vitro release revealed a sustained donepezil release over 4 h. Ex vivo drug permeation studies were conducted through porcine nasal mucosa, the results of which showed a high drug permeation for 4 h, achieving a permeation of about 80% of the initial drug amount.

Table 3 summarizes the most recent applications of microformulations as DDSs for delivering FDA-approved Alzheimer’s drugs, emphasizing the type of microformulation, their composition, the drug incorporated, the route of administration, and the primary outcomes in terms of DDS performance.

### 4.4. Nanoparticle-Loaded Hydrogel Systems

**Figure 8:**
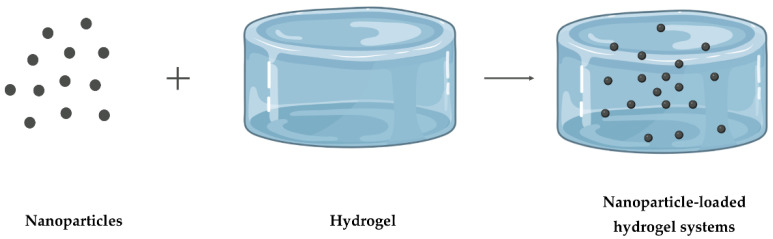
Schematic representation of the combination of loaded NPs and a hydrogel as DDS.

The major challenge in using NPs as a DDS is keeping the drug carrier for a desired time in the desired place. Drug release is more difficult to manage with hydrogels because it depends on the hydrogel’s constituents and the drug’s properties. The incorporation of NPs into hydrogels, as illustrated in Figure 8, has recently arisen as a novel DDS, combining their benefits to create new ones that neither could achieve alone [12].

Both NPs and hydrogels can individually enable multiple drug loading, improve drug bioavailability, and release drugs over time [8]. While the NPs can improve hydrogel mechanical properties by working as hydrogel cross-linking [8,100], the hydrogel shields the NPs from degradation and prevents their aggregation [101]. With the combination of NPs and hydrogels, it is possible to encapsulate more than one drug simultaneously, whether hydrophobic or hydrophilic [8,102]. In addition, the drug release kinetics is improved, and the initial burst release is avoided. The NLH systems create a depot at the administration site by combining the targeted NPs’ delivery with localized hydrogel delivery, allowing for prolonged local drug retention [103]. Furthermore, NLH systems can maintain the therapeutic levels of drugs, thus increasing their efficacy while minimizing systemic spread and toxicity [104]. Moreover, many studies have demonstrated the in vivo effectiveness of drug-loaded NPs combined with hydrogels for various biomedical applications [105].

When compared with each DDS individually, the integration of NPs into hydrogels has emerged to improve their performance. NLH systems have demonstrated the ability to regulate drug release and boost drug bioavailability while significantly decreasing drug administration frequency. It is possible to apply the NLH system locally and then provide a sustained release of the NPs in the site of action, allowing drug uptake in the required location. Numerous NLH systems have been exploited to solve the challenges associated with AD therapy. For example, Seo et al. (2020) developed a subcutaneously in situ-forming NLH system containing PLGA microspheres as a sustained release depot for donepezil [106]. The hydrogel was composed of hyaluronic acid-dopamine, reinforced with potassium phosphate to increase its retention capability. In vivo biodegradation and toxicity of the NLH were examined in mice after subcutaneous injection. The NLH offered a slower degradation rate and sustained drug release, which could reduce donepezil dose frequency and improve patient compliance. After subcutaneous administration of the NLH system, no acute toxicity was found in the animals, which was confirmed by histological examination of the vital organs.

**Figure 9:**
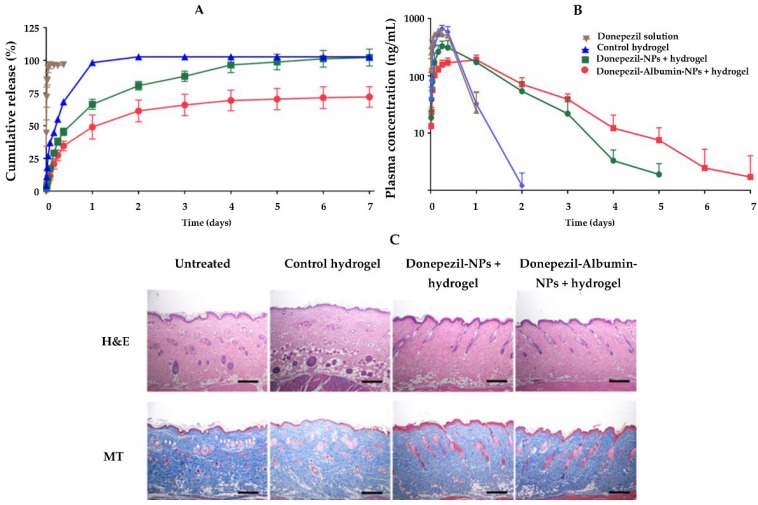
(**A**) In vitro release pattern of donepezil, (**B**) plasma concentration pattern over time, after in vivo administration of donepezil, and (**C**) skin histology 7 days after in vivo administration. The staining was performed with hematoxylin and eosin (H&E) and Masson’s trichrome (MT). Scale bar: 400 μm. Adapted with permission from [107], copyright © 2021 MDPI.

For the same purpose, Kang et al. (2021) also developed a subcutaneous approach for donepezil administration [107]. The DDS was an injectable hyaluronic acid hydrogel with donepezil-loaded lipid carriers incorporated. The hydrogel was additionally hybridized with human serum albumin to help with its sustained release by reducing the burst release of the drug. According to the findings, incorporating NPs in the hydrogel improved the rheological properties of the hydrogel. In addition, the NPs were found to retain the drug, and the final NLH system displayed a slower in vitro release profile than drug-loaded hydrogel and drug solution (Figure 9A). Sprague Dawley rats were subcutaneously injected with the drug-loaded hydrogel, drug-loaded NLH, and drug solution for in vivo pharmacokinetic and histological tests. Compared to controls, the plasma concentration (Figure 9B), mean residence time, and drug half-life in the animals administered with the NLH system were significantly higher. The histological study of the subcutaneous region where the NLH was injected revealed its retention for 7 days without symptoms of inflammation, demonstrating the NLH’s excellent biocompatibility (Figure 9C).

Mendes et al. (2019) also proposed NLCs incorporated in a hydrogel for a sustained transdermal release of donepezil for AD management [108]. The NLCs were prepared using the microemulsion technique, and various lipids were tested for their ability to increase donepezil skin penetration in vitro. The chosen formulation was composed of stearic acid, oleic acid, lecithin, and sodium taurodeoxycholate as a solid lipid, liquid lipid, surfactant, and co-surfactant, respectively. The hydrogel was prepared by adding Carbopol^®^ 940 to the NLC dispersion, followed by its neutralization using triethanolamine. The in vitro skin permeation studies were carried out with pig ear skin. The amount of donepezil that remained on the skin was less than 1% of the total drug administered to the skin, indicating a low risk of skin irritation. Additionally, the NLH system increased drug skin permeation, with NPs significantly boosting donepezil penetration through the skin. Due to NPs’ lipidic composition, they have a strong affinity for skin lipids, demonstrating their utility as transdermal carriers.

Al Harthi et al. (2019) prepared donepezil-loaded liposomes and incorporated them into a hydrogel. Various hydrogels were synthesized using gamma radiation with different polymers: PVP, chitosan, and thiolated chitosan [109]. The thiolated chitosan was chosen to integrate the liposomes because it was found to be less toxic and to have the optimum mucoadhesion and rheological properties for intranasal administration. Compared to drug-loaded liposomes in solution, drug-loaded liposomes embedded in the hydrogel retarded the in vitro release rate. The in vivo intranasal administration of the NLH system resulted in higher levels of the drug in both plasma and brain than the oral administration of the drug. Furthermore, the incorporation of drug-loaded liposomes into the hydrogel increased donepezil’s maximum plasma concentration by 46% when administered via the nasal route. The authors concluded that the NLH system created could significantly contribute to the effective transport of donepezil to the brain via the intranasal route.

Similarly, Rajput et al. (2022) developed donepezil-loaded liposomes incorporated in a hydrogel for intranasal administration [110]. In this case, the hydrogel was made up of gellan and xantham gum and had thermosensitive characteristics, allowing it to develop in situ. In vitro mucoadhesion, drug penetration capacity, and nasal ciliotoxicity studies were performed using sheep nasal mucosa. The interaction between formulation components and mucus of the nasal mucosa resulted in high mucoadhesive strength in the donepezil-loaded NLH. Permeation studies revealed that donepezil-loaded NLH had a higher drug permeation than donepezil-loaded hydrogel. No tissue damage or necrosis was observed, proving the safety of the NLH components, which was further supported by histopathological analysis. Additionally, the in vivo performance and the intranasal administration of the donepezil-loaded NLH system resulted in higher amounts of the drug in both blood and brain than the oral administration of donepezil. For donepezil-loaded NLH, there was less accumulation of donepezil in the remaining organs, implying less systemic dissemination. AChE levels in the rats’ brains significantly decreased, resulting in improved rat memory and cognitive impairments. The findings support the role of the NLH system in increasing the drug bioavailability and providing a direct path from the nose to the brain, resulting in lower systemic toxicity and adverse effects.

Transdermal applications of these NLH systems have also been explored for other AD drugs, such as rivastigmine, as proposed by Chauhan et al. (2019) [111]. As synergic approaches to treating dementia, the authors produced two hydrogels containing NLCs. The hydrogels were composed of two polymers: Eudragit^®^ E-100 and poly-butyl methacrylate-co-methyl methacrylate (PBMACMM), both containing rivastigmine-loaded NLCs. Compared to the commercially available transdermal rivastigmine patch, the authors found that loading the NPs in both hydrogels resulted in a more sustained in vitro release. In vivo, both hydrogels showed no sign of irritation, demonstrating the safety of the excipients. Animals treated with both NLH systems had higher drug levels in plasma and a longer and more consistent release pattern than those treated with conventional transdermal treatment. The increased bioavailability of rivastigmine and its sustained release obtained with the developed DDS have demonstrated their potential in improving AD treatment.

To improve nose-to-brain rivastigmine delivery, Cunha et al. (2022) proposed its encapsulation into NLCs and incorporated those NPs in a thermosensitive hydrogel [112]. The hydrogel was prepared with Pluronic^®^ F-127 and HPMC and optimized to obtain suitable properties for in situ solidification and delivery of loaded NPs. In vitro studies were performed to analyze the mucoadhesion in simulated nasal mucus, drug release, biocompatibility with nasal and pulmonary cells, and drug deposition in a nasal cast model. For the experiments, rivastigmine-loaded NLH was tested and compared to rivastigmine-loaded NPs, rivastigmine-loaded hydrogel, and rivastigmine solution as controls. Compared to controls, the rivastigmine-loaded NLH had higher nasal mucoadhesion and prolonged drug release. The studies revealed a concentration-dependent cytotoxicity effect in cells at higher NLH concentrations, while the systems were considered safe at lower concentrations. Finally, when compared to the NP solution, nasal deposition experiments demonstrated increased rivastigmine deposition in the olfactory region, which is the target deposition site for direct nose-to-brain delivery in the presence of the NLH system.

Other materials have been proposed for the intranasal administration of rivastigmine. Salatin et al. (2017) developed an in situ-forming hydrogel containing rivastigmine incorporated into NPs to overcome the toxicity issues of the drug. NPs were produced using two polymers: PLGA [113] and Eudragit^®^ RL-100 [114] and were then incorporated into the Pluronic^®^ F-127 hydrogel separately. The experimental parameters were optimized to obtain the ideal gelation time, temperature, stability, and mucoadhesive strength. Compared to drug-loaded hydrogel or drug-loaded NPs in solution, the authors found an improvement in rivastigmine’s in vitro release profile, with a sustained and controlled release for both drug-loaded NLHs. In vitro cytotoxicity and cellular uptake were performed with the drug-loaded NPs, unloaded NPs, and drug solution. PLGA-NPs had no cytotoxicity, whereas Eudragit^®^ RL-100 NPs have a dose and time-dependent cytotoxic effect against cells. PLGA-NPs showed a time-dependent increase in cellular uptake, while Eudragit^®^ RL-100 NPs showed a dose- and time-dependent increase. Rivastigmine permeability across sheep nasal mucosa was tested. While there were no significant changes in the permeability of PLGA-NP-loaded hydrogel, the Eudragit^®^ RL-100 NP-loaded hydrogel revealed higher rivastigmine permeability through the nasal mucosa. The authors concluded that the drug-loaded NLH can improve the therapeutic efficacy with a decrease in adverse effects, justified by the higher uptake of NPs by cells and the NLH’s ability to release the drug sustainably.

Table 4 summarizes the most recent NLH systems’ applications as DDSs for delivering FDA-approved Alzheimer’s drugs. The table briefly provides the different characteristics of the NLH systems developed, such as NP composition, hydrogel composition, their loaded drug, route of administration, and leading in vitro and in vivo outcomes.

## 5. Discussion and Conclusions

Aside from the fact that the majority of pharmacological treatments for AD merely address the symptoms of the disease, they also have drawbacks that restrict their complete efficacy. The drugs’ weak pharmacodynamic and pharmacokinetic parameters prevent them from reaching the brain in effective therapeutic concentrations. The patient’s compliance is also compromised by the side effects of the high doses administered and the need for frequent administration. Additionally, AD causes memory loss and behavioral changes that interfere with daily medication intake. Thus, the development of strategies to boost the effectiveness of FDA-approved Alzheimer’s drugs while reducing their side effects and compliance issues is required. Potential therapeutic approaches for AD have increased as a result of developments in the field of nanomaterials. Hence, a promising strategy may come from DDSs that can be produced with the appropriate properties to enhance the physicochemical properties of the drug in the biological environment and overcome the biological barriers [16]. In the context of treating AD, the DDS could effectively direct the drugs to the brain by improving their transport across the BBB [15]. DDSs can also guarantee a controlled and sustained drug release with fewer side effects.

Herein, we reviewed and discussed the recently developed DDS as a strategy to increase the efficacy of FDA-approved Alzheimer’s drugs. NPs received the most research attention (46% of all papers herein discussed). The findings revealed that NPs could release drugs sustainably over time and improve their bioavailability, and pharmacokinetic and pharmacodynamic properties. The drugs were successfully delivered across the BBB, resulting in increased concentrations in the target tissue. In this manner, NPs employed as DDSs attempted to avoid unwanted side effects and compliance questions. The NPs most often employed were polymeric ones, which accounted for around 37% of the reported works. Due to their biocompatibility, biodegradability, and ability to provide a prolonged and controlled release, these NPs have demonstrated promising outcomes for AD therapy both in vitro and in vivo. PLGA nanocarriers, which made up more than 50% of the reported polymeric NPs, were used most often. The second most often employed NPs were liposomes, accounting for 32% of the reported works. The key advantages of these nanocarriers are their nontoxicity, non-immunogenicity, biocompatibility, biodegradability, and ability to encapsulate drugs with various lipophiles and molecular weights [53]. In addition, liposomes possess a high versatility since it is possible to change their size, membrane fluidity, and surface characteristics to adapt their behavior to the biological environment [115,116,117,118]. The lipid NPs account for 21% of the papers reported herein, followed by organic NPs and dendrimers, with 5% each.

Some authors have also employed hydrogels and hydrogel-forming microneedles as DDSs for long-term AD therapy. Based on the studies reported in this review, using hydrogels and hydrogel-forming microneedles as DDSs corresponds to about 7% and 12% of all the developed DDSs. These DDSs could boost drug bioavailability, prolong their release, and maintain the drug dosages over an extended period. Thus, this DDS has the potential to minimize drug delivery frequency while also improving patients’ compliance. In addition, compared to commercialized transdermal patches, the hydrogels herein described revealed reduced skin irritation. The advantage of microneedles, comparable to hydrogels, is their ability to penetrate the skin and overcome the skin barrier properties.

There is broad research on microparticles, microspheres, and microemulsions; however, few preparations contain FDA-approved Alzheimer’s drugs. These types of DDSs comprise 12% of the works herein described, corresponding to five formulations. The presented microformulations improved drugs’ bioavailability and pharmacokinetic behavior, boosted brain drug levels, and prolonged plasma drug levels.

The synergistic effects of NLHs in drug delivery have recently been investigated to overcome the individual drawbacks of NPs and hydrogels. NLHs were investigated in about 22% of the works herein discussed. In these systems, the most-used NPs were lipidic NPs, accounting for about 44% of the reported studies. Polymeric NPs and liposomes came in second and third, with 33% and 23% of the studies, respectively. The NLH systems are complex materials that have attracted considerable attention for drug delivery purposes. These DDSs combine the benefits of hydrogels and NPs in a single drug delivery platform, enabling the achievement of different functions in one administration [18]. In addition, the incorporation of NPs into hydrogels considerably increases the drug loading capability, which can boost the therapeutic effects.

Beyond the DDSs herein reported, there are other cutting-edge DDSs that, though not demonstrated for AD drug delivery, hold excellent potential, such as micelles. Micelles are core–shell carriers made of amphiphilic block copolymers that self-assemble into specific arrangements in an aqueous solution. These DDSs are particularly interesting for drug delivery applications, with several reported works for managing AD [119,120,121]. Both hydrophobic and hydrophilic drugs can be enclosed in the core–shell structure, which comprises a hydrophobic core and a hydrophilic shell. Micelles can successfully protect drugs from degradation, and the shell characteristics may hinder reticuloendothelial system identification and bloodstream elimination. The size and morphology of the micelles can also be easily regulated, and surface modification with specific molecules is simple [122].

**Figure 10:**
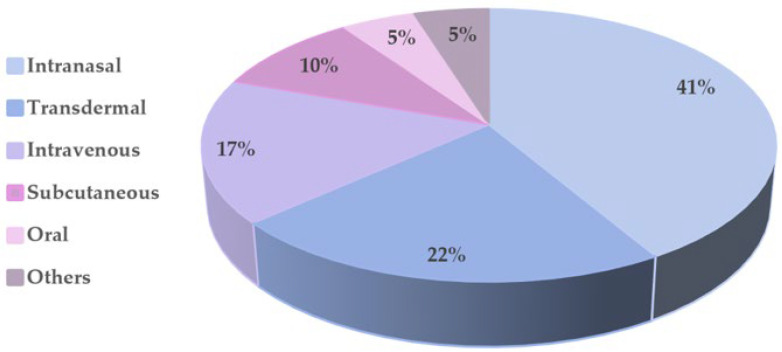
Schematic distribution of the developed DDS as a strategy to improve the efficacy of FDA-approved Alzheimer’s drugs by their administration routes. The graph was created based on the works reported in this review.

As demonstrated in Figure 10, the majority of the studies mentioned in this work were designed for intranasal administration, where the drugs are directly delivered into the brain through the olfactory mucosa, overcoming the BBB [15].

Except for intranasal administration, the BBB hinders the therapeutic effectiveness of the remaining administration routes (including transdermal, intravenous, subcutaneous, and oral) [18]. As a result, the surface of the NPs must be modified with targeting molecules to increase their capacity to overcome the BBB. NPs’ transportation by endocytosis mediated by specific receptors is the most relevant mechanism for transporting molecules across the BBB. The appropriate selection of the ligand is crucial to increase the transport efficiency of NPs through the BBB, since the receptor must be expressed at the target site. Of the reviewed studies, only two developed a brain targeting DDS by functionalizing NPs’ surfaces with brain-targeting molecules. Functionalized NPs allow for very selective drug transport across the BBB, as demonstrated by Topal et al. (2021), using APOE, and Gothwal et al. (2019), using lactoferrin [64,79]. Two other works reported coating NPs’ surfaces with polysorbate 80 to increase their stability and successfully transport them across the BBB [62,77].

Not all the works herein reviewed have tested the DDS’s efficacy in vivo. Therefore, future in vivo studies of those DDSs are still required and may reveal details regarding their drug delivery capability, systemic efficacy, safety, and toxicity in the biological environment. Most of the reviewed works evaluated some pharmacokinetic and pharmacodynamic parameters of the developed DDS in vivo to determine the drug concentrations in blood and brain at different time points, including areas under the curve for blood and brain, maximum drug concentrations in blood, and times elapsed to reach these concentrations. However, specific parameters related to brain targeting efficacy provide crucial information about DDS behavior and should be considered and assessed before being translated into clinical studies. Those parameters include the maximum drug concentrations in the brain and times elapsed to reach these concentrations, the ratio of the drug in the blood and brain at predetermined timepoints, drug targeting efficiency (how much of the drug accumulates in the brain), and drug transport percentage (the percentage of a drug that enters the brain via direct routes; for example, through the intranasal route) [123]. The analysis of these parameters will provide insights into the drug targeting efficiency, the appropriate dosage concentrations, and administration route. Despite the abundance of promising preclinical evidence for several DDSs, and although the reviewed DDS tested in vivo are predicted to improve the efficacy of the FDA-approved Alzheimer’s drugs, none reached the clinical trials phase. Therefore, in the near future, an evaluation of the safety and efficiency of those DDSs through human clinical trials could result in promising and affordable AD treatment approaches.

However, even though the DDSs could improve the efficacy of FDA-approved Alzheimer’s drugs, they could not revert the disease or stop the progression of the disease. Only the recently approved drug Aduhelm™ is intended to treat the disease. Several others are undergoing clinical trials for AD treatment, as summarized in Table 5. These investigations aim to target some leading causes of AD, such as amyloid peptide, tau protein, or neuroinflammation. Ongoing Phase III clinical trials include antibodies against Aβ (Donanemab, Gantenerumab, Lecanemab, and Solanezumab) and a vaccine (UB-311). Phase II clinical trials focusing on tau protein are testing a vaccine (ACI-35) and a small molecule (LY3372689). In addition, currently in Phase II clinical trial, there is an antibody to treat neuroinflammation (Pepinemab) and a small molecule to target Aβ (Simufilam). The ACI-35, for example, is a vaccine to elicit an immune response targeted to pathological conformers of phosphorylated tau, whose formulation is based on liposomes [124]. Therefore, in the future, DDSs can also be combined with these new therapeutic candidates for AD to increase their efficacy.

## Figures and Tables

**Table 1:** The most recent applications of NPs as DDSs for delivering FDA-approved Alzheimer’s drugs.

Drug	NPs Type	NPs Composition	Route of Administration	Main Outcomes	Ref.
Donepezil	Liposomes	Carboxymethyl cellulose, DSPC, cholesterol, and PEG	Intranasal	Sustained release of donepezil and enhanced bioavailability in the plasma and brain using liposomes.	[60]
	Polymeric	Chitosan	Intranasal	NPs improved the pharmacokinetic properties and bioavailability of the drug, increasing its concentration in the target tissue.	[61]
		PLGA	Intravenous	NPs significantly increased drug transport to the brain, resulting in higher drug concentration in the target tissue.	[62]
		PLGA-PEG	Intravenous	Donepezil was successfully delivered across the BBB by NPs and released in a controller manner.	[63]
	SLNs	Dynasan^®^ 116	Intravenous	NPs exhibited a sustained release of the drug, a higher uptake by cells, and increased permeability.	[64]
Galantamine	Liposomes	Soya phosphatidylcholine, cholesterol, and PG	Intranasal	Liposomes could effectively deliver galantamine by the nose-to-brain route with superior pharmacokinetic behavior and enhance AChE inhibition.	[65]
	Polymeric	PLGA	Intravenous	NPs provided a sustained release of the drug compared to galantamine solution and are predicted to boost therapeutic effects and reduce side effects.	[66]
	SLNs	Glyceryl Behenate	Oral	SLNs enhanced the bioavailability of the drug, modulated its time course in vivo, and provided a controlled release.	[67]
	Polymeric	Chitosan	Intranasal	The pharmacodynamic behavior of the drug was enhanced by NPs. The animals given the NPs had higher AChE levels and recovered significantly from induced amnesia	[68]
Rivastigmine	Polymeric	MPEG-PCL	Intravenous	NPs were able to delay the drug release and increase the in vivo brain uptake clearance of rivastigmine, which translated into improved memory deficit.	[69]
		Chitosan	Intranasal	NPs provided a controlled and sustained release of the drug, with superior brain targeting efficiency than rivastigmine solution.	[70]
	Liposomes	Soya lecithin and cholesterol	Intranasal	Liposomes improved the pharmacokinetic and pharmacodynamic parameters of the drug. Drug-loaded liposomes reversed the memory deficit characteristic of AD compared to the free drug.	[71]
		PEG-DSPE, Lecithin, DDAB, and Tween^®^ 80	Intranasal	Liposomes prolonged the release of rivastigmine and improved its bioavailability. The drug levels in both plasma and brain were increased about fourfold.	[72]
		Phosphatidylcholine, Dihexadecyl phosphate, cholesterol, and glycerol	Subcutaneous	Liposomes provided a sustained and controlled release of the drug. The use of nanocarriers also resulted in significantly improved cognitive impairment and increased AChE activity.	[73]
		Cholesterol, Lecithin, oleic acid, Labrafil^®^, Labrasol^®^, Pluronic^®^ F-127, PG, and PEG	Transdermal	Liposomes enhanced rivastigmine permeation through the skin and maintained plasma levels within the therapeutic window after topical application.	[74]
	SLNs	Glyceryl Behenate	Intranasal	SLNs provided higher in vitro and ex vivo nasal permeation of the drug. The nasal mucosa remained intact, proving its safety for intranasal administration.	[75]
		Glyceryl monostearate	Intranasal	The pharmacokinetic drug profile, bioavailability, and drug concentration in plasma and the brain were improved by SLNs in vivo.	[76]
	Organic NPs	Silica	Intravenous	NPs allowed for a sustained release in vitro and improved the drug pharmacokinetics parameters.	[77]
Memantine	Polymeric	PLGA	Oral	NPs prolonged the drug release, which reduces the frequency of oral administration. In vivo, memantine-loaded NPs improved learning abilities and reduced β-amyloid brain plaques and inflammation associated with AD.	[78]
	Dendrimers	PAMAM	Intravenous	Dendrimers improved the pharmacokinetic parameters of the drug. The DDS revealed significant improvement in behavioral responses and memory in vivo.	[79]

**Table 2:** The most recent applications of hydrogels as DDSs for delivering FDA-approved Alzheimer’s drugs.

Drug	Hydrogel Composition	Route of Administration	Main Outcomes	Ref.
Galantamine	Methacrylated gelatin	Intracerebroventricular injection	Galantamine administration via hydrogel was found to be effective in reducing Aβ aggregation while also enhancing neuroinflammation, antioxidant activity, and neuronal development.	[82]
Donepezil	PVA and PVP	Transdermal	The hydrogel enhanced drug bioavailability and increased its plasma levels, allowing for long-term maintenance of drug doses.	[83]
	Poloxamer 407 and Poloxamer 188	Intranasal	The hydrogel improved drug bioavailability and targeting efficiency, resulting in more effective drug delivery to the brain.	[84]

**Table 3:** The most recent applications of microformulations as DDSs for delivering FDA-approved Alzheimer’s drugs.

Microformulation Type	Drug	Microformulation Composition	Route of Administration	Main Outcomes	Ref.
Microneedles	Donepezil	Hydrogel base: carboxymethyl cellulose Microneedles: HPMC	Transdermal	The microneedles efficiently transported donepezil across the skin, and the DDS increased drug bioavailability.	[87]
		Microneedles: PMVE/MAH, PEG, sodium carbonate Drug reservoir: PVP and glycerol	Transdermal	In vitro, the DDS significantly improved drug penetration through the skin. Drug plasma concentrations in vivo were considerably higher than the control.	[88]
		Hydrogel base: Polydimethylsiloxane Microneedles: PVP	Transdermal	The hydrogel improved the drug’s bioavailability and enabled its sustained release through the skin. The DDS has the potential to minimize drug delivery frequency while also improving patient compliance.	[89]
	Memantine	Microneedles: PDMS and alumina Drug reservoir: PLA	Transdermal	The DDS was well tolerated by the skin and was able to deliver memantine transdermally for 3 days.	[90]
	Rivastigmine	Hydrogel base and microneedles: alginate and k-carrageenan	Transdermal	Compared to commercially available drug patches, the new DDS was safer and did not cause skin irritation. When the DDS was employed, the drug was released in a more efficient and controlled manner.	[92]
Microparticles	Rivastigmine	Ethanol, water, and l-leucine	Inhalation	Spray-dried microparticles presented suitable physicochemical characteristics, aerodynamic properties, and aerosolization performance for rivastigmine inhalation delivery.	[94]
		SAIB, PLGA, NMP, and Pluronic^®^ F-68	Intramuscular	Microparticles extended plasma levels of rivastigmine and increased its bioavailability.	[95]
Microspheres	Rivastigmine	Ethyl cellulose, chitosan, and PVA	Intranasal	The DDS significantly improved memory retention, biochemical parameters, and drug pharmacodynamics in vivo. The modification of the microsphere surface improved its mucoadhesion.	[96]
Microemulsion	Rivastigmine	Glyceryl caprylate, labrasol, transcutol-P, and water	Intranasal	Mucoadhesive microspheres increased the drug’s bioavailability and brain concentration following intranasal administration.	[98]
	Donepezil	Castor oil, labrasol, transcutol-P, PG, and water	Intranasal	The microspheres were able to release donepezil in a sustained manner. In ex vivo nasal mucosa, a high permeability was likewise attained.	[99]

**Table 4:** The most recent applications of NLH systems as DDSs for delivering FDA-approved Alzheimer’s drugs.

Drug	NPs Type	NPs Composition	Hydrogel Composition	Route of Administration	Outcome	Ref.
Donepezil	Polymeric	PLGA	Hyaluronic acid	Subcutaneous	The NLH system provides a sustained release of the drug, which can minimize donepezil dose frequency and enhance patient compliance.	[106]
	NLCs	Stearic acid and oleic acid	Hyaluronic acid	Subcutaneous	The NLH system revealed a biodegradation time of roughly 7 days and a sustained release of the drug, making it a viable option for a local depot with long-term drug release.	[107]
		Stearic acid, oleic acid, lecithin, and sodium taurodeoxycholate	Carbopol^®^ 940	Transdermal	The addition of drug-loaded NPs to the hydrogel resulted in a sustained release and a significant increase in drug penetration into the skin.	[108]
	Liposomes	Cholesterol and dipalmitoylphosphocholine	Thiolated chitosan	Intranasal	Donepezil-loaded NPs combined into a hydrogel could give a long-lasting release, as well as significantly boost drug levels in the blood and brain.	[109]
		Hydrogenated soy phosphatidyl choline and cholesterol	Gellan gum and xanthan gum	Intranasal	Intranasal administration of the NLH increased drug levels in the target tissue due to a direct conduit from the nose to the brain, reducing systemic toxicity. The AChE activity was decreased, alleviating cognitive impairments.	[110]
Rivastigmine	NLCs	Glycerol monostearate and castor oil	Eudragit^®^ E-100 and PBMACMM	Transdermal	Rivastigmine-loaded NLH enhanced skin permeation of the drug, achieving higher drug levels in plasma compared to conventional rivastigmine transdermal therapy.	[111]
		Precirol^®^ 5 ATO, and Vitamin E	Pluronic^®^ F-127 and HPMC	Intranasal	The NLH system provided a prolonged drug release and higher nasal mucoadhesion, increasing drug retention time. The results highlight the potential of the DDS to improve nose-to-brain delivery.	[112]
	Polymeric	PLGA	Pluronic^®^ F-127	Intranasal	A high cellular uptake of NPs by cells was obtained, which may provide an enhanced therapeutic efficacy in vivo with a decrease in side effects.	[113]
		Eudragit^®^ RL-100	Pluronic^®^ F-127	Intranasal		[114]

**Table 5:** New therapeutic drug candidates in clinical trials for AD.

Drug	Target	Mechanism of Action	Route of Administration	Sponsor	FDA Status	Clinical Trial Identifier	Ref.
ACI-35	Tau	A liposome-based vaccine to elicit an immune response targeted to pathological conformers of phosphorylated tau.	Intramuscular	AC Immune SA (Lausanne, Switzerland), Janssen (Belcey, Belgium)	Phase II	NCT04445831	[124]
Donanemab	Amyloid	An antibody designed to bind to a pyroglutamate form of Aβ that is aggregated in amyloid plaques.	Intravenous	Eli Lilly & Co. (Indianapolis, IN, USA)	Phase III	NCT04437511	[125]
Gantenerumab	Amyloid	An antibody designed to bind to Aβ fibrils.	Subcutaneous	Roche (Basel, Switzerland)	Phase III	NCT04374253	[126]
Lecanemab	Amyloid	An antibody designed to bind to Aβ protofibrils.	Intravenous	Biogen, Eisai Co., Ltd. (Tokyo, Japan)	Phase III	NCT03887455	[127]
LY3372689	Tau	Inhibitor of the O-GlcNAcase enzyme.	Oral	Eli Lilly & Co. (Indianapolis, IN, USA)	Phase II	NCT05063539	[128]
Pepinemab	Inflammation	Antibody to semaphorin 4D, a multifunctional membrane glycoprotein expressed by oligodendrocytes and astrocytes in the CNS.	Intravenous	Vaccinex, Inc. (New York, NY, USA)	Phase I/ II	NCT04381468	[129]
Simufilam	Amyloid	Molecule designed to bind to filamin, a protein that stabilizes Aβ-42 and the α7 nicotinic acetylcholine receptor (reported to trigger tau phosphorylation).	Oral	Cassava Sciences (Austin, TX, USA)	Phase II	NCT04079803	[130]
Solanezumab	Amyloid	An antibody directed against the mid-domain of the Aβ peptide.	Intravenous	Eli Lilly & Co. (Indianapolis, IN, USA)	Phase III	NCT00905372	[131]
UB-311	Amyloid	Synthetic peptide vaccine generated N-terminal anti-Aβ antibodies, which neutralized Aβ toxicity and promoted plaque clearance.	Intramuscular	United Neuroscience (Dublin, Ireland)	Phase III	NCT02551809	[132]

## Data Availability

Not applicable.
